# The New Dental Graduate

**Published:** 1921-08

**Authors:** Eugene S. Talbot

**Affiliations:** Chicago, Ill.


### THE NEW DENTAL GRADUATE

BY EUGENE S. TALBOT, M.D., D.D.S., CHICAGO, ILL.

Here is to the health of the new dental graduate. I love him, I have great respect for him, but I feel sorry for him. He entered the dental college four years ago with the belief that when he graduated he would be able to enter practice and successfully conduct his professional duties. The same thought prevailed one hundred years ago when a jeweler or a blacksmith entered the office of a dentist and remained for six months or a year and then started out to conquer the world. The same thought prevailed fifty years ago when I graduated from a dental school after two years of college training. I believed I was capable of doing justice to my patients because I was a born mechanic. I had served my time as an apprentice in a machine shop and had become a master mechanic and foreman in the Pennsylvania Railroad shops at nineteen. I had taken first prize in college for my mechanical ability—my plate work was always exibited for years to the students to show what a first course student could accomplish. I, therefore, entered upon practice with considerable assurance. I had been in practice only four years when I discovered that while mechanics were necessary, a successful practice required histology, pathology, and later, bacteriology.

The same question confronts you, my confrères, today. One hundred years ago, fifty years ago and today, all practitioners were good mechanics for the period. One hundred years ago there were no dental colleges. Fifty years ago there were perhaps eight or ten dental schools. Today there are fifty-four schools in the United States and Canada.

Fifty years ago only two years were required for graduation. Today four years are required. The same conditions exist today in regard to practice as they did eighty years ago, when the first dental school was established. As time goes on and we become better informed it is not mechanics that we require as much as histology, pathology, bacteriology and a broad university education to know what has been accomplished, to think, to reason, to concentrate, to be able to add fact to fact, and then by studious effort add more facts to the sum total of the literature of our profession.

The majority of you have only a high school education. Some have only a common school education. None of you have received the training in histology, pathology or bacteriology necessary to practice your specialty successfully. The reason for this is that the medical side of dentistry has progressed from year to year far beyond the progress in our dental schools. Today you are entering a profession which is entirely ignorant of the medical side of dental practice.—Unwarranted criticism; Ed. Register.

Having pointed out to you the weak side of your professional training and what the profession now requires, the object of this paper is to try and direct your future training so that you may become a successful practitioner. You will no doubt practice your profession for the next fifty years. The practice of dentistry today will not be the practice of the specialty ten years hence. The dental practitioner then will be a medical graduate. He will not only be well grounded in the general principles of medicine, but he will also be a university graduate. You will have become too old to compete with him. Even today physicians are selecting medical graduates for consultants in obscure pathologic conditions of the mouth.

At this writing, March 8th, I am attending the meeting of the Council on Medical Education, which convenes here in Chicago every year at this time. I enjoy these meetings because the papers are presented by the best educated men in the country on up-to-date subjects. The discussion at this meeting was confined to "Symposium on Graduate Training in the Various Medical Specialties." Each specialty in medicine, except dentistry, was represented by a paper in which all agreed that two to three years should be devoted to special study after graduation in medicine to perfect one in the calling he wished to practice. This would apply to dentistry as well as other specialties.

Every graduate in dentistry must decide now whether he wishes to excel in his practice, become an ordinary tooth carpenter, or sink to the level of a laboratory man. If he wishes to excel he must continue his studies for the next three years. He can not take a full medical course of five years for want of time and money.. He can, however, take a correspondence course and educate himself equal to a university graduate. He can also assist in forming study classes in his community and take up histology, pathology, bacteriology and perfect himself equal to a medical graduate.

Never in the history of dentistry have such great changes taken place as will occur in the next ten years, and those who graduate from dental schools today will be the sufferers when the great change in education takes place.

This is no idle talk, for the handwriting is already on the wall and I can foresee from my past experience what the future has in store for us.

When you start in practice, associate with men (physicians) who are better educated than you are. I adopted this plan and it works out well.

Undertake a few things and carry them out to the highest possibilities. Attend medical societies as well as dental. Read medical books and journals as well as dental. Attain the respect of the medical profession and you will attain success.—*Oral Hygiene*, for May.

